# Isolation and Functional Analyses of a Putative Floral Homeotic C-Function Gene in a Basal Eudicot London Plane Tree (*Platanus acerifolia*)

**DOI:** 10.1371/journal.pone.0063389

**Published:** 2013-05-15

**Authors:** Jiaqi Zhang, Zhineng Li, Cong Guo, Guofeng Liu, Manzhu Bao

**Affiliations:** Key Laboratory of Horticultural Plant Biology, Ministry of Education, College of Horticulture and Forestry Sciences, Huazhong Agricultural University, Wuhan, Hubei, P.R. China; Wuhan Botanical Garden, Chinese Academy of Sciences, China

## Abstract

The identification of mutants in model plant species has led to the isolation of the floral homeotic function genes that play crucial roles in flower organ specification. However, floral homeotic C-function genes are rarely studied in basal eudicots. Here, we report the isolation and characterization of the *AGAMOUS* (*AG*) orthologous gene (*PaAG*) from a basal eudicot London plane tree (*Platanus acerifolia* Willd). Phylogenetic analysis showed that *PaAG* belongs to the C- clade *AG* group of genes. *PaAG* was found to be expressed predominantly in the later developmental stages of male and female inflorescences. Ectopic expression of *PaAG*-*1* in tobacco (*Nicotiana tabacum*) resulted in morphological alterations of the outer two flower whorls, as well as some defects in vegetative growth. Scanning electron micrographs (SEMs) confirmed homeotic sepal-to-carpel transformation in the transgenic plants. Protein interaction assays in yeast cells indicated that PaAG could interact directly with PaAP3 (a B-class MADS-box protein in *P. acerifolia*), and also PaSEP1 and PaSEP3 (E-class MADS-box proteins in *P. acerifolia*). This study performed the functional analysis of *AG* orthologous genes outside core eudicots and monocots. Our findings demonstrate a conserved functional role of *AG* homolog in London plane tree, which also represent a contribution towards understanding the molecular mechanisms of flower development in this monoecious tree species.

## Introduction

The molecular mechanism of flower development has been the subject of intensive investigation over the last two decades. Initially, the classical ‘ABC’ model of flower development was proposed [1]–[2], and this has subsequently been expanded to the ‘ABCDE’ model [3]–[5]. In this latter model, five classes of floral homeotic function genes are recognized as responsible for determining floral organ identity by acting in a combinatorial fashion. Thus, class A and E genes control the formation of sepals, class A, B and E genes together control the formation of petals, class B, C and E genes act together to determine stamen identity, class C and E genes specify carpel identity, and class D genes act with the class E genes to specify ovule identity. With the exception of the *APETALA2* (*AP2*) class A orthologous genes, all floral homeotic function genes in the ‘ABCDE’ model are members of the MADS-box transcription factor family [6]–[7].

*AGAMOUS* (*AG*) is a floral homeotic C-function gene in *Arabidopsis thaliana*, and is necessary for specification of stamens and carpals, as well as for floral meristem determinacy [8]–[9]. Flowers with strong *ag* mutant phenotypes show the complete homeotic conversion of stamens into petals and carpels into sepals. These mutant lines also show indeterminacy of the floral meristem. Studies of the *Arabidopsis ag ap2* double mutant suggest that *AG* also promotes the specification of ovule identity [10]. Functional characterization of *AG* orthologous genes has been conducted in many plant species and, in general, these studies indicate a conserved functional role in flower development [11]–[14]. In contrast with this, C-function genes are rarely studied in basal eudicots.

We are specifically interested in the genes regulating flower development in London plane tree (*Platanus acerifolia*), a hybrid of American sycamore (*P*. *occidentalis*) and Oriental plane (*P*. *orientalis*), and also a basal eudicot [15]. Hybrid vigour of the trees has contributed to London plane becoming an important city landscape tree in the great majority of temperate and subtropical regions. However, a drawback of this tree species for urban application may be its reproductive characteristics. London plane is a monoecious tree, i.e. with separate male and female inflorescences on the same plant. The development of both male and female flowers is initiated in the middle of May, approximately one year before anthesis occurs. Flowers are borne on capitula which are formed inside the petioles, together with the leaf buds. During April of the following year, the matured male and female flowers begin to open. Following flower opening, a large quantity of pollen is released which may cause pollinosis and seasonal asthma in the human population [16]–[17]. Seed hairs released from the dehiscent cones later in the year may also pollute urban environments and detrimentally influence human health. Thus, the breeding of sterile lines of London plane tree would greatly improve its application value. The aim of our present study is to isolate and characterize the key genes from London plane tree involved in flower development, with a view to exploiting them for molecular breeding purposes. In addition, these studies would further broaden our knowledge and understanding of flower development in *Platanus*.

In this article, we report the isolation of the *AG* orthologous gene *PaAG* from London plane tree. Expression analyses, functional characterization and protein interaction assays were performed. The putative functional role of this *AG* orthologue in flower development is discussed. The potential suitability of such floral homeotic function genes as targets for the bioengineering of male and/or female sterility is discussed.

## Materials and Methods

### Plant Material and Growth Conditions

Plant materials for gene cloning and real-time quantitative PCR analysis were obtained from variously aged London plane trees (namely, one year-old and four year-old juvenile individuals, and a 40 year-old mature tree), all of which were field-grown in Huazhong Agricultural University, Wuhan, China. Male inflorescences at various ontogenetic stages were collected on May 25^th^, June 16^th^, July 15^th^, August 15^th^, October 16^th^ and December 15^th^ of the first developmental year (2007), and March 15^th^ and April 16^th^ of the second developmental year (2008). Female inflorescences at various ontogenetic stages were collected on June 16^th^ and December 15^th^ of the first year (2007) and April 16^th^ of the second year (2008). Subpetiolar buds from adult and juvenile trees were all collected on December 15^th^, 2007. Samples were frozen in liquid nitrogen immediately after collection and stored at −80°C until used. Total RNA was isolated using the improved CTAB (cetyl trimethyl ammonium bromide) method described by Li *et al.* [18].

For genetic transformation experiments, the day-neutral tobacco (*Nicotiana tabacum*) cultivar ‘Xanthi’ was used as the host plant. Transformed tobacco plants were grown in a containment greenhouse with a 14 h photoperiod and a 25°C/17°C day/night temperature.

### Isolation of the *AG*-orthologous Gene and Sequence Analyses

Cloning of the *Platanus AG*-orthologous gene was performed using a strategy of 3′/5′-RACE (rapid amplification of cDNA ends), as we have described previously [19]. The degenerate primers PaAGF and PaAGR, used to amplify partial coding sequences of the putative *AG*-orthologous gene, were designed according to the conserved MADS domain and K domain, respectively. The cycling program consisted of an initial denaturation at 94°C for 3 min, followed by 35 cycles at 94°C for 30 s, 58°C for 30 s, 72°C for 30 s, and a final extension of 72°C for 5 min. Gene specific primers PaAGrF and PaAGrR were used to amplify the 3′ and 5′ terminal regions, respectively, of *AG*-orthologous gene. Universal 3′ and 5′ PCR primers were supplied by the SMART™ cDNA Library Construction Kit (Clontech, Palo Alto, CA, USA). The RACE conditions were 94°C for 3 min, followed by 5 cycles at 94°C for 25 s, 67°C for 30 s, 72°C for 1 min; followed by 5 cycles at 94°C for 25 s, 65°C for 30 s, 72°C for 1 min; followed by 25 cycles at 94°C 25 s, 63°C for 30 s, 72°C for 1 min, and a final extension of 72°C for 5 min. The complete cDNA sequences of *AG*-orthologous gene were obtained using the primer pairs PaAGvF and PaAGvR with Pfu DNA Polymerase (Stratagene, La Jolla, CA). The PCR conditions were 94°C for 3 min, followed by 32 cycles at 94°C for 30 s, 60°C for 30 s, 72°C for 1 min, and a final extension of 72°C for 5 min. All primer pairs are described in Table S1. The amplified products corresponding to the expected size were cut from the agarose gel, cloned in the pMD18-T vector (Takara, Japan), and sequenced using the universal M13 forward primer.

### Phylogenetic Analyses

The complete deduced amino acid sequence of PaAG was used for phylogenetic analysis. The multiple sequence alignment was performed using software ClustalX 1.83. Sequences were imported into MEGA 4 software for phylogenetic analysis and the phylogenetic tree was constructed using Neighbor Joining (NJ) analyses [20]. Bootstrap values were derived from 1,000 replicate runs. A total of 26 MADS-box amino acid sequences were obtained from GenBank (http://www.ncbi.nlm.nih.gov). The accession numbers of the sequences used are as follows: *Akebia trifoliata* AtrAG-1 (AAT46102); *A. thaliana* AG (NP_567569), SEP3 (NP_001185081), SHP1 (P29381), SHP2 (P29385) and STK (NP_192734); *Citrus unshiu* CitMADS1 (BAF34911); *Corylus avellana* CaMADS1 (AAD03486); *Gossypium hirsutum* GhMADS3 (AAL92522); *Lycium barbarum* LbSEP3 (ADP09004); *Malus domestica* MdMADS15 (CAC80858); *Mangifera indica* MiSEP3-L (AEO45959); *Meliosma dilleniifolia* MdAG1 (AAS45686); *N. tabacum* NAG1 (AAA17033); *Oryza sativa* OsMADS3 (AAA99964); *Petunia hybrida* FBP7 (CAA57311) and FBP11 (CAA57445); *Populus tremuloides* PtSEP3 (AAO49811); *Populus trichocarpa* PtAG1 (AAC06237); *Prunus persica* PPERSTK (ABQ85556) and PpMADS4 (AAU29513); *Theobroma cacao* TcAG (ABA39727); *Triticum aestivum* WAG (BAC22939); *Vitis vinifera* VvMADS1 (AAK58564) and VvMADS4 (AAM21344); *Zea mays* ZAG1 (AAA02933).

### Expression Analyses by Real-time Quantitative PCR

To determine the expression pattern of *PaAG*, real-time quantitative PCR was performed. RNA samples were pre-treated with RQ1 RNase-Free DNase (Promega, USA) in order to eliminate all non-RT-dependent background. 3 µg of DNase pre-treated total RNA was reverse transcribed in a total volume of 20 µL with 0.5 µg oligo(dT)_15_, 0.5 mM dNTPs, 10 mM DTT, 40 U RNasin® Ribonuclease Inhibitor (Promega, USA) and 200 U SuperScript II RNase H- reverse transcriptase (Invitrogen, CA, USA).

Primers (PaAGtF and PaAGtR, see Table S1) for real-time quantitative PCR were designed within the nonconservative C terminal region and tested to ensure that amplification of a single discrete band occurred, with no primer-dimer products. Reactions were performed with the SYBR Premix Ex Taq (Takara, Japan) and analyzed in the ABI Prism 7000 Sequence Detection System (Applied Biosystems, USA). Real-time quantitative PCR products were amplified using 0.5 µl of template from the RT reaction mixture, 5 µl 2×SYBR Green Master Mix, 0.5 µl forward and reverse primer (10 µmol/µl), and water to a final volume of 10 µl. PCR amplification employed a 10 min denaturing step at 95°C, followed by 40 cycles at 95°C for 15 s and 60°C for 1 min. The levels of gene expression were calculated by ABI Prism 7000 Sequence Detection System Software (Applied Biosystems, USA) and normalized with the results of *TPI* (*Triose phosphate isomerase*). Real-time quantitative PCR was performed in three replicates for each sample and data are shown as mean values ± SD (standard deviation).

### *PaAG* Expression Vector Construct and Transformation

The full-length cDNA sequence of *PaAG-1* was amplified with primers PaAGvF and PaAGvR (Table S1), and cloned in the pMD18-T vector. Sequence accuracy and insertion direction were confirmed by sequencing. After digestion with *Sal*I and *Kpn*I restriction enzymes, the insert was subcloned into the modified binary vector pBI121 containing the CaMV*35S* promoter and the *Nos* terminator, to create the construct *35S::PaAG-1.*

*Agrobacterium tumefaciens* EHA105 containing the expression vector was used to transform tobacco (*N. tabacum*) cultivar ‘Xanthi’. Transformation of the tobacco lines was performed as described by Horsch *et al* [21]. Transgenic lines were verified by genomic amplification of the transgene using a primer to the *35S* promoter (35SF) and a *PaAG*-specific primer (PaAGvR) (Table S1).

### Scanning Electron Micrograph (SEM) Observation

Floral organs were fixed with 2% glutaraldehyde for approximately 24 h. After washing in a 0.1 M cacodylate buffer, samples were dehydrated in a graded ethanol series, dried in a desiccator (HCP-2; Hitachi), and coated with a film of gold. Observations were carried out using a JSM-6390LV scanning electron microscope (NTC, Japan).

### Yeast Two-hybrid Screen

The yeast strain AH109 was used. Yeast cells were transformed by the LiAc/DNA/PEG method according to the Yeast Protocols Handbook from Clontech (http://www.clontech.com). The full length coding sequence of *PaAG-1* (containing the *Nde*I and *Bam*HI restriction sites at the 5′ and 3′ ends, respectively) was amplified by PCR from the sequenced clones using the primers PaAG-yF and PaAG-yR. The PCR products were introduced in-frame with the GAL4 activation domain in the pGADT7-Rec vector, and with the GAL4 binding domain in the pGBKT7 vector. The ORFs of *P. acerifolia SEPALLATA1* (*PaSEP1*) and *SEPALLATA3* (*PaSEP3*) (containing the *Eco*RI and *Bam*HI restriction sites generated by PCR) were introduced in-frame with the GAL4 activation domain in the pGADT7-Rec vector. The primers used are described in Table S1. The construction of *PaPIs*-pGBKT7 and *PaAP3*-pGADT7-Rec has been described previously [19]. Co-transformed yeast cells were tested for interaction/activation on selective SD media without leucine, tryptophan, histidine and adenine, respectively.

## Results

### Molecular Cloning and Sequence Analysis of *P. acerifolia AG* Orthologous Genes

In order to isolate the putative floral homeotic C function gene of *P. acerifolia*, degenerate primers were designed within the conserved MADS domain and K domain through nucleotide alignments of various known *AG* orthologous gene sequences. A 456 bp cDNA segment was obtained using this primer pair. Sequence alignment indicated that it was an *AG* orthologous segment. We named it *Platanus acerifolia AGAMOUS* (*PaAG*). Using this gene segment, 3′ and 5′-RACE reactions were conducted to obtain the 3′ and 5′ terminal regions of *PaAG*, respectively. A 202-bp 5′ UTR (untranslated region) and a 243-bp 3′ UTR with a poly(A) tail were obtained. Gene-specific primers within the 5′ and 3′ UTRs were used to amplify the full length *PaAG* cDNA sequence. Analysis of *PaAG* sequences revealed that the genome of *P*. *acerifolia* contains at least two *PaAG* gene copies, *PaAG*-*1* and *PaAG*-*2*. The gene copies were found to differ at two nucleotide sites located at ORF (open reading frame) positions 331 (T/C) and 672 (A/T). However, these two *AG* orthologous genes encode the same PaAG protein. Such a small difference at the nucleotide level implied that they may be the alleles inherited from the two parent species, *P. orientalis* and *P. occidentalis*. The sequences of *PaAG*-*1* and *PaAG*-*2* have been deposited in the GenBank library under accession numbers JX855925 and JX855926.

**Figure 1:**
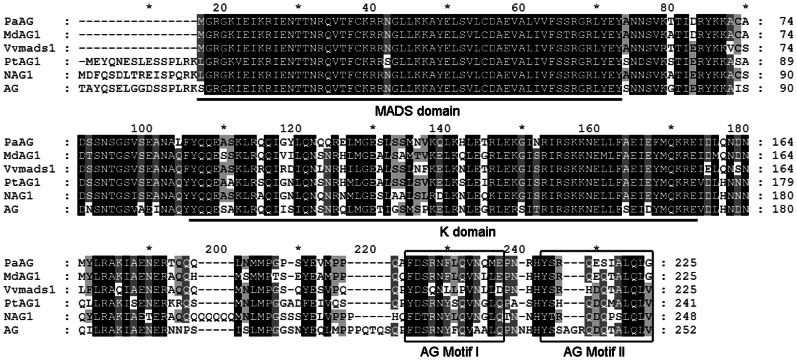
A comparison of the deduced amino acid sequences of PaAG and other AG homologs. Sequences of AG homologs from *A*. *thaliana* (AG), *N*. *tabacum* (NAG1), *Meliosma dilleniifolia* (MdAG1), *Vitis vinifera* (Vvmads1) and *Populus trichocarpa* (PtAG1) were aliged with PaAG. Identical amino acid residues in relation to PaAG are black and conserved residues are in grey. Dashes denote gaps inserted to maximize alignment. The conserved MADS domain and K domain are underlined. The AG motifs I and II in the C terminal region are boxed.

**Figure 2:**
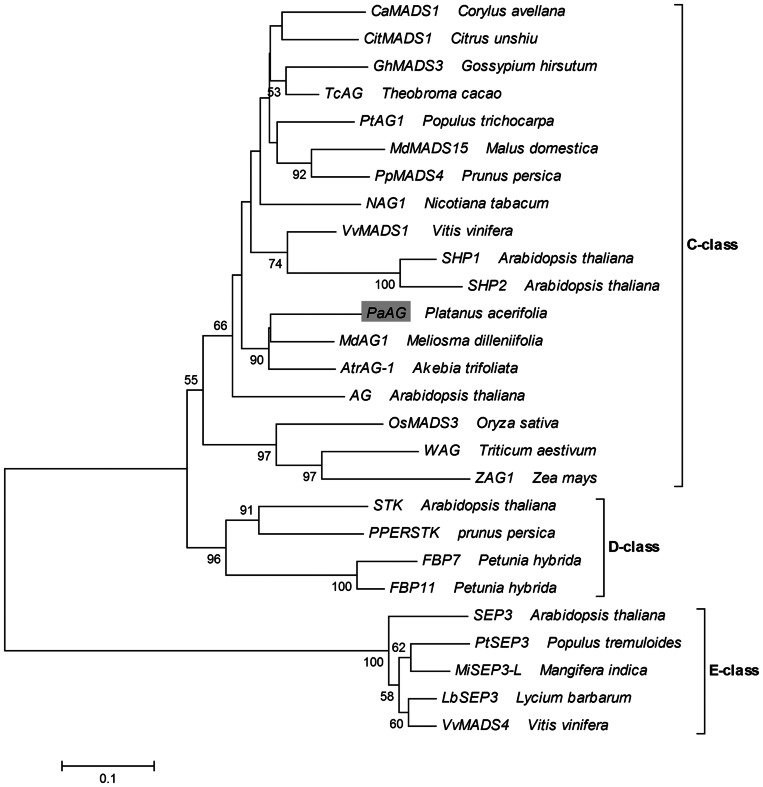
Phylogenetic analysis of *AG* homologous genes. Phylogenetic tree was calculated from the deduced amino acid sequence of PaAG and other plant AG homologs using the neighbor-joining method. PaAG appears inside a gray box. The different MADS-box classes are indicated at the right. The bootstrap confidence values (%) from 1000 replicates are indicated on the branches. The scale indicates the average substitutions per site.

*PaAG*-*1* and *PaAG*-*2* encode a putative protein of 225 amino acid residues, which shares high identity with other AG-related proteins (Fig. 1). Like other Type II MADS box proteins, PaAG possesses a highly conservative MADS domain and a relatively well-conserved K (keratin-like) domain. Furthermore, the conserved AG motifs I and II are present at the C-terminal region of PaAG [22]. Phylogeny reconstruction was performed using the predicted amino acid sequence of PaAG and 26 other MADS box proteins. The various AG-related genes were divided into three major clades (representing the C-, D- and E-class MADS-box genes) in the neighbor-joining tree. Phylogenetic analysis (Fig. 2) confirmed the placement of *PaAG* within the clade of C-class MADS-box genes, supported by a high bootstrap value. The results also showed that *PaAG* was closely related to *MdAG1* and *AtrAG-1*, which were also isolated from basal eudicots.

### Expression of *PaAG* in London Plane Tree Organs

To examine the expression profile of *PaAG* in London plane tree, real-time quantitative PCR was carried out using cDNA derived from various vegetative and reproductive tissues, as taken from juvenile and adult trees.

**Figure 3:**
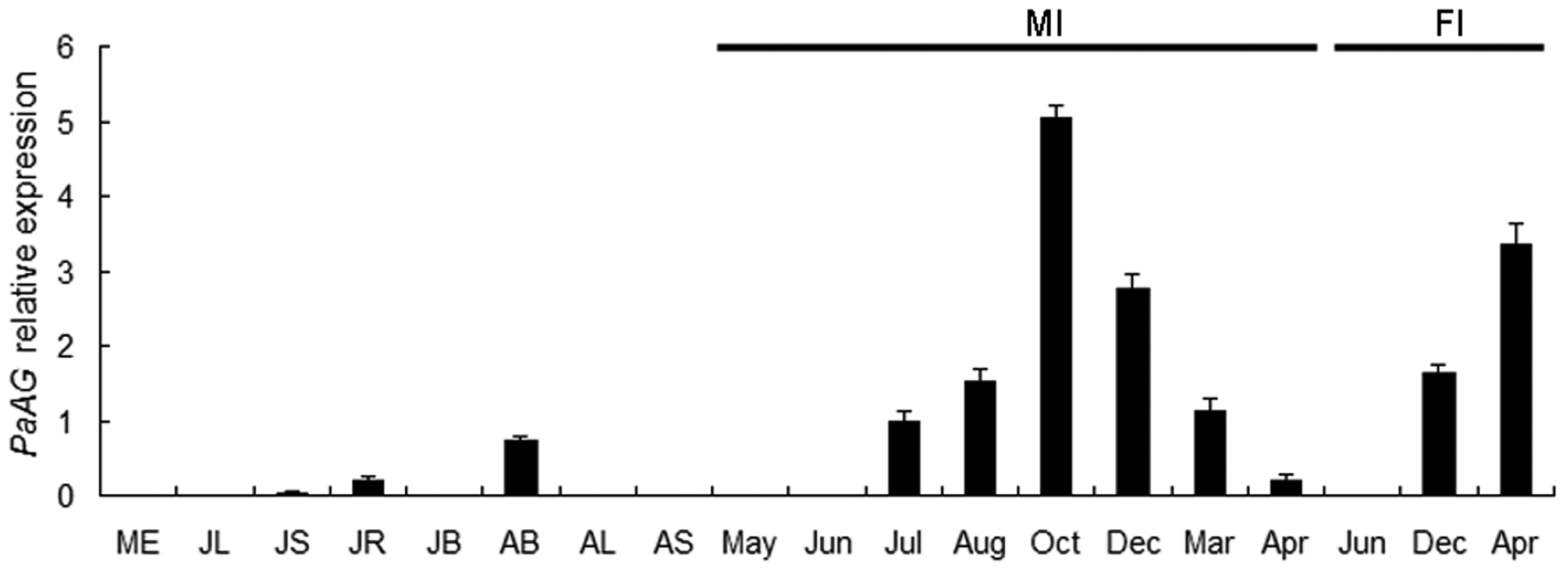
Expression analyses of *PaAG* transcripts in London plane tree. Relative mRNA levels were determined using real-time quantitative PCR and normalized against the reference gene *TPI*. Values are mean ± SD of three replicates. ME = mature embryos; JL = leaves of one year-old seedlings; JS = stems of one year-old seedlings; JR = roots of one year-old seedlings; JB = subpetiolar buds of four year-old juvenile tree; AB = subpetiolar buds of the adult tree; AL = leaves of the adult tree; AS = stems of the adult tree; MI = male inflorescences (collected in May, June, July, August, October, December, March and April); FI = female inflorescences (collected in June, December and April).

We found that *PaAG* was expressed in the stems and roots of juvenile trees, albeit at extremely low levels. *PaAG* transcripts were also detected at moderate levels in subpetiolar (leafy) buds from the adult tree. However, no expression was detected in other vegetative organs or in mature embryos (Fig. 3).

In most plant species, the primary role of floral homeotic C function genes is in flower development, in particular in defining stamen and carpel identity [23]–[24]. Thus, we conducted detailed examination of the expression of *PaAG* in male and female inflorescences sampled at different developmental stages. The development of the male and female inflorescences of London plane tree takes place over a period of almost one calendar year, from bud initiation in May of the first year to opening of the mature flower in April of the following year. Expression analysis revealed that *PaAG* transcripts were not detected in the very young inflorescences collected during May-June of the first year. However, *PaAG* transcripts were detected at significant levels during the later developmental stages of both male and female inflorescences. Relative expression levels reached a peak in male inflorescences collected during October of the first year (i.e. approx. 5-fold higher expression levels than detected in young male inflorescences collected during July). Following this peak, expression of *PaAG* gradually diminished, and only very weak expression was detected in the mature male inflorescences collected in April of the following year. In contrast to the trend seen in the male inflorescences, expression of *PaAG* in female inflorescences remained relatively high in the mature flowers collected in April, i.e. approx. 17-fold higher than the expression levels seen in the male inflorescences sampled at the same stage (Fig. 3).

### Ectopic Expression of *PaAG* in Tobacco

London plane trees have a long juvenile period, typically of 8–10 years. Clearly, this will significantly delay the phenotypic observation of transgenic manipulation of a mature plant trait such as flowering. Thus, in order to rapidly gain information about *PaAG* function in flower development, we employed a heterologous transformation system. Twenty-two independently transformed tobacco plants containing the full-length sense *PaAG-1* driven by the 35S promoter were generated. The presence of the transgene in these lines was confirmed by PCR of the genomic DNA. Ten of the transformed lines showed various degrees of phenotypic alteration in the reproductive organs, as compared to wild-type plants.

**Figure 4:**
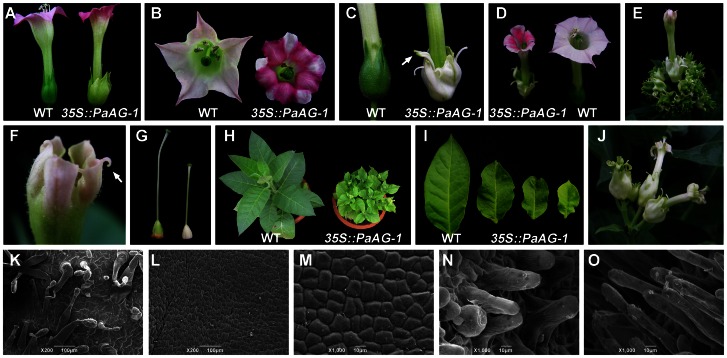
Comparison of the phenotypes of wild-type and *35S::PaAG-1* transgenic tobacco plants. (A) Wild-type (left) and weak phenotype *35S::PaAG-1* flowers (right). (B) Petals from a wild-type (left) and a weak phenotype *35S::PaAG-1* flower (right). (C) Sepals from a wild-type (left) and a strong phenotype *35S::PaAG-1* flower (right). Arrow indicates the stigma-like structure at the tip of the sepal. (D) Comparison of a strong phenotype *35S::PaAG-1* flower (left) and a wild-type flower (right). (E) Inflorescence of a strong phenotype *35S::PaAG-1* line. (F) Close inspection of the outwards curved filament-like structures (arrow shown) from a strong phenotype *35S::PaAG-1* flower. (G) The fourth whorl organ of a strong phenotype *35S::PaAG-1* flower (right) compared with a wild-type carpel (left). (H) Comparison of a wild-type plant and a strong phenotype *35S::PaAG-1* line. (I) Leaves of strong phenotype *35S::PaAG-1* lines (right three) compared with a wild-type leaf (left). (J) Inflorescence of a T1 generation of the strong phenotype *35S::PaAG-1* line. (K)∼(O) SEM observation of the outer face of floral organs from wild-type and strong phenotype *35S::PaAG-1* plants. (K) Wild-type sepal. (L) *35S::PaAG-1* sepal. (M) Wild-type carpel. (N) stigma-like structure from the tip of the sepal of *35S::PaAG-1*. (O) Wild-type stigma.

Four of these lines displayed a moderate phenotype, in which it was noted that they all developed sepal organs with a pale green/loose morphology, in contrast to the dark-green/compact form of sepals seen in wild-type plants (Fig. 4A). In addition, petal pigmentation was altered in these transgenic lines, i.e. flowers contained various patches of pink and white coloration, in contrast to the uniform light pink of the wild-type petals (Fig. 4B). Six of the transgenic lines displayed a strong floral phenotype. In these lines, morphology of the first and second whorls of flower organs was significantly changed. Unlike wild-type flowers, sepals of strong phenotype lines appeared white, and there was a marked reduction in the number of trichomes on the sepal surfaces. In addition, the tips of the sepals developed green stigma-like structures (Fig. 4C). This homeotic sepal-to-carpel transformation phenotype was confirmed by SEM observation. The epidermal cells of the sepals in wild-type plants could be described as being shaped like ‘jigsaw pieces’, and they had an irregular arrangement, and were covered with numerous trichomes and stomata (Fig. 4K). By contrast, epidermal cells of the first whorl organs in the strong phenotype lines were essentially rectangular in shape, conformed to a more regular arrangement, and had relatively low numbers of trichomes and stomata (Fig. 4L); thus, they appeared more similar to the epidermal cells of the wild-type carpel wall (Fig. 4M). The surfaces of the stigmatic tissues found at the sepal tips of the transgenic plants possessed numerous cylindrical projections (Fig. 4N), and these, too, were reminiscent of the outer face of the true stigma tissues of wild-type plants (Fig. 4O).

The sizes of the flower tubes and limbs were reduced in strong phenotype lines. Petals in these lines had deep clefts, whereas petals in wild-type flowers were connately fused (Fig. 4D). Furthermore, petals of some of the *35S::PaAG-1* tobacco flowers developed anomalous outwardly curling tips (Fig. 4E and F). However, SEM observation revealed that the microstructure of epidermal cells from organs of the second whorl was not significantly different between transgenic and wild-type (control) flowers (data not shown). The ovary organs in the strong phenotype lines appeared white, as opposed to the green wild-type forms (Fig. 4G). Apart from this effect, however, no morphological or microstructure alterations were detected in the third and fourth whorl organs of the *35S::PaAG-1* flowers. The presence of the *PaAG-1* transgene could also impact on vegetative growth. Thus, some of the tobacco plants with strong flower phenotypes were significantly shorter than wild type plants of the same age. Leaves in these lines were also small and contorted (Fig. 4H and I). Two strong phenotype lines were allowed to self-fertilize. Seed set in the two lines was much reduced compared to wild-type plants. The offspring displayed similar phenotypic traits to the parent plants (Fig. 4J).

### Interaction Behaviors of B-, C- and E-class MADS-box Proteins from *P. acerifolia*

**Figure 5:**
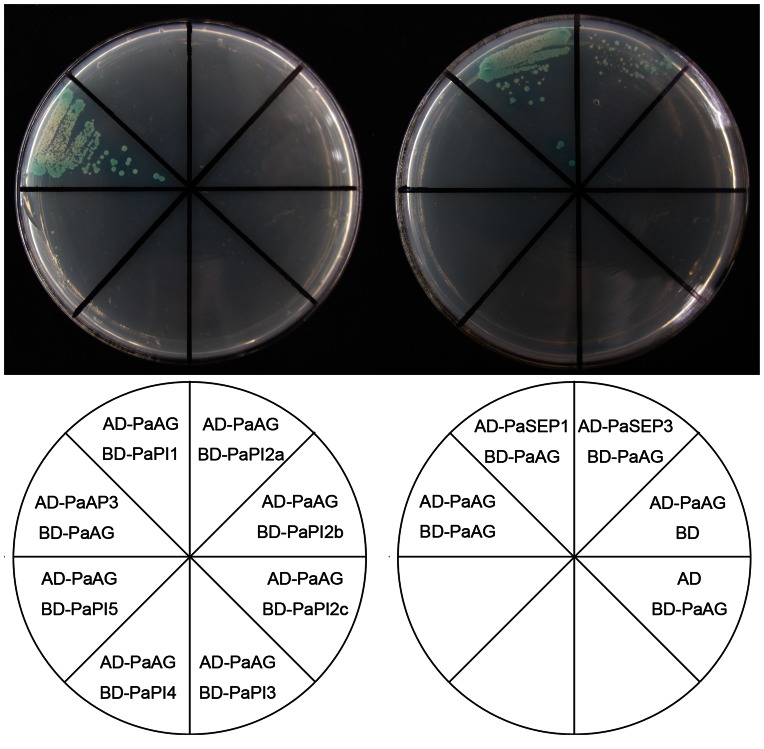
Yeast two-hybrid interactions between PaAG and the London plane B−/E-class MADS-box proteins. The chart below shows bait (BD) and prey (AD) plasmids. Two microliters of the respective yeast cultures were plated onto a selection medium without adenine, histidine, leucine and tryptophan.

In the ABCDE model of floral development, B+C+E class genes specify stamen identity, and C+E class genes together define carpel identity [5]. We wished to determine whether the product of *PaAG* is capable of forming the appropriate molecular interactions to be involved in stamen and carpel development. Therefore, yeast two-hybrid assays were performed between PaAG and the B- and E- class MADS-box proteins from *P. acerifolia*. We have previously reported the isolation of five *PI* homologous genes from London plane, corresponding to seven different *PaPI* transcripts (i.e. *PaPI1*, *PaPI2a*, *PaPI2b*, *PaPI2c*, *PaPI3*, *PaPI4* and *PaPI5*) [19]. Protein interaction assays within yeast cells revealed that PaAG could not interact with any of these PaPI B-class proteins. By contrast, PaAG could interact with another B-class MADS-box protein namely, PaAP3 (the APETALA3 ortholog in *P. acerifolia*; GenBank Accession No. EF488452). Furthermore, PaAG could also interact with the two E-class MADS-box proteins PaSEP1 (the *SEPALLATA1* ortholog in *P. acerifolia*; GenBank Accession No. GU296507) and PaSEP3 (the SEPALLATA3 ortholog in *P. acerifolia*; GenBank Accession No. EF686225). PaAG proteins did not form homodimers in the yeast cells (Fig. 5).

## Discussion

The results of several different analyses support the conclusion that the gene we have named as *PaAG* is, indeed, the *P. acerifolia* C-class *AG* orthologue. Firstly, the deduced amino acid sequence of PaAG revealed high identity with other AG-related proteins. The highly conserved motifs specific to *AG* homologues, i.e. AG motifs I and II [22], were also found at the end of the C-terminal region of PaAG. Gene duplication events and subfunctionalization have occurred in the *AG* subfamily, and this C-class family has also given rise to the D-class function gene [22]. Thus, some of the *AG*-like genes belong to the D-lineage, eg. the D-class gene *SEEDSTICK* (*STK*; formerly *AGL11*) [25]–[26]. In this study, phylogenetic analysis confirmed the placement of *PaAG* within the *C*-lineage with a high bootstrap value support. These results indicated that *PaAG* was associated with known C-function *AG* homologues, rather than with floral homeotic D-function genes.

In *Arabidopsis*, *AG* expression is confined to the flower organs of the third and fourth whorls [9]. In studies of other species, *AG* orthologous genes are also expressed in vegetative tissues, although the expression levels are significantly lower than in reproductive organs [27]–[28]. Such observations are consistent with our findings for *PaAG* in London plane tree. Thus, expression analyses revealed that *PaAG* transcripts could be detected in vegetative tissues (i.e. stems and roots from the juvenile tree, and subpetiolar buds from the adult tree), but the levels were relatively low compared to those detected in male and female inflorescences. In London plane tree, male and female inflorescences develop almost simultaneously. The levels of *PaAG* transcripts varied in male and female inflorescences which had been collected from different developmental stages. According to our previous observations of histological sections [15], both male and female inflorescences begin to differentiate during the middle of May in a typical year. Similarly, the sepal and petal organs begin to differentiate in the middle of June. *PaAG* transcripts were undetectable in such young male and female inflorescences (i.e. samples collected in May and June of the first year of bud emergence). These results indicate that the expression *of PaAG* may not correlate to sepal and petal development. In another unisexual species, namely silver birch (*Betula pendula* Roth), expression of the *AG* homologue *BpMADS6* is also found to be very weak, or even undetectable, in the early developmental stages of male and female inflorescences [29]. An *AG* homologue in *V. vinifera*, *Vvmads1*, is expressed only in the later stages of flower development [30]. From early July, when the stamen and pistil primordia appear, flowers of the London plane tree enter the stamen and pistil differentiation phase. *PaAG* was found to be expressed to relatively high levels in both male and female inflorescences collected from July, onwards. Expression of *PaAG* continued into April of the following year, when the male and female flowers were fully matured. A comparison of male and female inflorescences, collected at equivalent developmental stages, revealed that the largest difference occurred during April, when the expression of *PaAG* was approx. 17-fold higher in the female compared to male inflorescences. We conjecture that this differential expression may relate to the biological roles of *PaAG*. According to studies in *Arabidopsis* and *Petunia*, *AG* orthologous genes are responsible for directing the formation of stamens and carpels, and also acts in combination with D- and E-class genes to promote ovule identity [5], [10], [14], [31]. *Platanus acerifolia* female inflorescences collected during April of the second developmental year had completed morphological differentiation, but the ovules were still undergoing maturation. Thus, the high expression level seen in female inflorescences at this stage indicates that *PaAG* may be involved in ovule development in London plane tree as well. Indeed, the *AG* orthologous genes of many species have been reported to be highly expressed in the ovules [14], [27], [32], [33]. Organ differentiation within male flowers of *P. acerifolia* was found to be almost complete by October of the first year. The period from October through to the following April was mainly associated with the development of the male gametophytes. Declining expression levels of *PaAG* throughout the late developmental stages of male inflorescences indicates that *PaAG* is unlikely to be involved in the processes of pollen maturation. Studies in poplar (*P. trichocarpa*) have also revealed that expression of the *AG* homologs *PTAG1/2* decreases in male flowers following differentiation of the stamen primordia [27]. Unfortunately, the extremely small size of the flowers on the capitula of London plane tree precluded us from separating the individual floral whorls for RT-PCR analysis. We were also unable to successfully perform *in situ* hybridization due to the dense hairs covering both male and female flowers. Therefore, the detailed expression pattern of *PaAG* in flower organs could not be determined in this study. Nevertheless, the observed expression profile of *PaAG* within male and female inflorescences is broadly consistent with the gene having a role as the *AG* homologue of *P. acerifolia*.

In order to further assess *PaAG* function, we employed a heterologous expression system. The constitutive expression of *PaAG*-*1* within tobacco resulted in the homeotic transformation of sepal organs into carpelloid structures. Morphological alterations were also detected in the flower organs of the second whorl, namely the petals of the transgenic plants were small and had deep clefts, and filament-like structures were observed at the petal tips. These phenotypic changes are similar to those reported in tobacco following the ectopic expression of *AG* orthologous genes from silver birch and cotton [29], [34]. As well as changes in the development of reproductive organs, strong phenotype plants of *35S::PaAG*-*1* tobacco also displayed changes in vegetative growth, notably small curled leaves and reduced plant stature. The literature reports that transgenic *Arabidopsis* plants overexpressing functional *AG* orthologues show similar vegetative growth defects [12], [13], [35], [36]. Thus, the results of functional analyses in the tobacco host are consistent with the conclusion that *PaAG* is a floral homeotic C function gene.

According to the floral quartet model, C-class genes, together with the B- and E-class genes, are required for petal identity. In addition, C-class genes together with the E-class genes, are responsible for carpel identity. The combinatorial action of these floral homeotic genes is based on protein interaction [5], [31], [37], [38], [39]. We investigated the potential for *in vivo* protein interaction between PaAG and the *P*. *acerifolia* B- or E-class proteins using yeast cell assays. The results confirmed that PaAG could directly interact with PaAP3, PaSEP1 and PaSEP3. Inferring similar interaction behaviors *in situ* within London plane tree further supports the viewpoint that *PaAG* is a functional C-class gene.

We have previously reported the cloning of a number of MADS-box genes in *P. acerifolia*, and some of these have been shown to fulfill a similar functional role to the analogous *Arabidopsis* floral organ identity genes [15], [19], [40]. Characterization of floral homeotic function genes could help to reveal the molecular mechanisms underlying flower development in London plane tree. Furthermore, these floral organ identity genes are possible choices for the manipulation of flower development. There are precedents for the application of such genes in studies of plant sterility [29], [41], [42], [43], which thereby provide good reference points for the future success of similar approaches to the breeding of male and/or female sterile lines in London plane tree.

## Supporting Information

Table S1 **A list of primers used in this study.**
(DOC)
