# Supplementary material for: Isolation and Functional Analyses of a Putative Floral Homeotic C-Function Gene in a Basal Eudicot London Plane Tree (Platanus acerifolia)
Source: PLoS One. 2013 May 15;8(5):e63389. doi: 10.1371/journal.pone.0063389 (PMC3655187; doi:10.1371/journal.pone.0063389)
Supplement: Table S1 — A list of primers used in this study. (DOC) [file pone.0063389.s001.doc]

**Table S1** A list of primers used in this study.

| Pimer | Sequence (5′ - 3′) | Description |
| --- | --- | --- |
| PaAGF | AGGATHGAGAACWCAACDAATCG | degenerate |
| PaAGR | TCMAGYTSAATCTCCCTTTTCTG | degenerate |
|  | (D=ATG, H=ACT, M=AC, S=CG, W=AT, Y=CT) |  |
| PaAGrF | GGGTATCTCCAAAACCAGCAGAGG | 3′ RACE |
| PaAGrR | CGCAGTTTGGAGGCTTCTTGCTGGT | 5′ RACE |
| PaAGvF | TCTTCCATCCTCCCTGATTTTCTC | expression vector construct |
| PaAGvR | GCAATCTCTGGTTCTTATCGCTCTC | expression vector construct |
| PaAGtF | AGGGAGATTGA TTTGCAGAACGATA | real-time quantitative PCR |
| PaAGtR | GCGGTTGGGCTCCATTTGATTTACTTG | real-time quantitative PCR |
| PaTpiF | GCCACAACAAGGATTATTTATGGAG | tpi control |
| PaTpiR | TGATTCCCAAGTTTAAGCACTCTTC | TPI control |
| PaAG-yF | GCTCATATGATGGGAAGGGGAAAGATCGAGAT | yeast two-hybrid assay |
| PaAG-yR | GATGGATCCTTAACCTAGCTGGAGGGCGATTG | yeast two-hybrid assay |
| PaSEP1-yF | AGTGAATTCATGGGGAGAGGACGAGTTGAACT | yeast two-hybrid assay |
| PaSEP1-yR | GATGGATCCTCAAAGCATCCACCCAGGAATGA | yeast two-hybrid assay |
| PaSEP3-yF | AGTGAATTCATGGGGAGAGGTAGGGTTGAGTTG | yeast two-hybrid assay |
| PaSEP3-yR | GATGGATCCTCAAGCTAACCATACTGGCATGTAAT | yeast two-hybrid assay |
| 35SF | ACGCACAATCCCACTATCCTTC | CaMV 35S promoter |
